# Host upregulation of lipid droplets drives antiviral responses

**DOI:** 10.15698/cst2021.09.256

**Published:** 2021-08-25

**Authors:** Ebony A. Monson, Karla J. Helbig

**Affiliations:** 1 School of Life Science, La Trobe University, Melbourne.

**Keywords:** lipid droplets, interferon, innate immunity, organelle, virus, antiviral

## Abstract

When a host cell is infected by a virus, it activates the innate immune response, setting off a cascade of signalling events leading to the production of an antiviral response. This immune response is typically robust and in general works well to clear viral infections, however, viruses have evolved evasion strategies to combat this, and therefore, a better understanding of how this response works in more detail is needed for the development of novel and effective therapeutics. Lipid droplets (LDs) are intracellular organelles and have historically been thought of simply as cellular energy sources, however, have more recently been recognised as critical organelles in signalling events. Importantly, many viruses are known to take over host cellular production of LDs, and it has traditionally been assumed the sole purpose of this is to supply energy for viral life cycle events. However, our recent work positions LDs as important organelles during the first few hours of an antiviral response, showing that they underpin the production of important antiviral cytokines following viral infection. Following infection of cells with either RNA viruses (Zika, Dengue, Influenza A) or a DNA (Herpes Simplex Virus-1) virus, LDs were rapidly upregulated, and this response was also replicated following stimulation with viral mimic agonists. This upregulation of LDs following infection was transient, and interestingly, did not follow the well described homeostatic mechanism of LD upregulation, instead being controlled by EGFR. The cell's ability to mount an effective immune response was greatly diminished when inhibiting EGFR, thus inhibiting LD upregulation during infection, also leading to an increase in viral replication. In this microreview, we extrapolate our recent findings and discuss LDs as an important organelle in the innate immune response.

## LIPID DROPLETS ARE UPREGULATED DURING PATHOGEN INFECTION

How are LDs induced? The most well-described mechanism of LD biogenesis involves the budding off of LDs from the endoplasmic reticulum membrane, initiated by the hydrolysis of fatty acids by the enzyme phospholipase A (PLA). LDs alternate between periods of growth and depletion, with the accumulation of LDs being linked to numerous disease sates (obesity, fatty liver disease, cardiovascular disease, diabetes, and numerous cancers). The induction of LDs specifically from pathogen infection is not a new phenomenon, with the first observation of this occurring in the 1800s in a *Mycobacterium leprae* infection. The accumulation of LDs from viral infection, however, is relatively new and until recently, the biogenesis mechanisms of LDs from pathogen infection has not been well described. Our study identified that virally induced LDs have an alternate biogenesis mechanism to the ordinary homoeostatic LD biogenesis. EGFR has previously been shown to elevate LD numbers in human colon cancer cells and we found that both EGFR and PI3κ, but not PLA_2_ were the driving force behind the induction of LDs following viral infection. Virally driven LDs were blocked by inhibition of PI3K/mTOR pathways, supporting their dependency on selected upstream pathways, fitting with our findings that EGFR engagement plays a role in the induction of virally induced LDs. We found that this mechanism underpinned LD accumulation in infections of both RNA and DNA viruses, however, the mechanisms driving LD accumulation in bacterial infections are still unknown, and it is plausible that there are different receptors mediating this response.

## LIPID DROPLETS CONTRIBUTE TO AN ANTIVIRAL RESPONSE

The upregulation of LDs has been linked with multiple infections; however, it wasn't until recently that LDs were also discovered to play a role in early host responses. Our recent work demonstrated that the upregulation of LDs following a viral stimulus plays an antiviral role in the cell, where this upregulation contributes to a heightened type I and III interferon response *in vitro*. To understand if LD accumulation during viral infection could influence the immune response of the infected cell, we treated cells with the fatty acid, oleic acid, prior to infection with Zika virus or HSV-1. Increasing the number of LDs prior to infection resulted in an enhanced interferon response of the infected cells, and lead to a significant decrease in viral mRNA. This response was reversed when EGFR was blocked, and the cell's ability to upregulate LDs was inhibited. Although the upregulation of LDs is now known to increase the production of antiviral IFNs, the mechanism for this is not well understood, and still requires further investigation.

**Figure 1:**
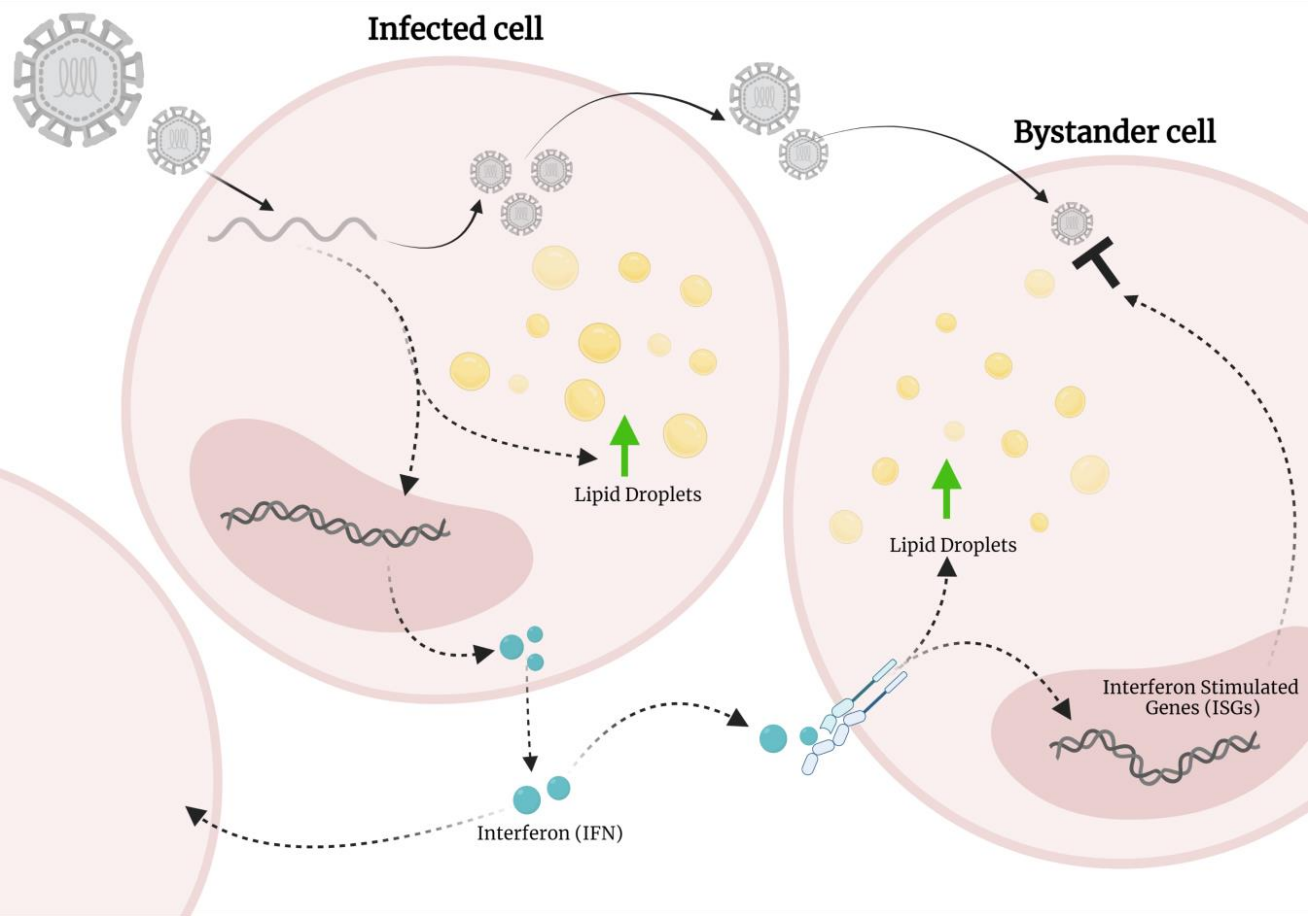
FIGURE 1: Lipid droplets upregulated by IFN may contribute to a bystander effect. Viral infection of a cell leads to the increase of lipid droplets (LDs) and the production of interferon. Interferon is secreted out of cells where it sets up a bystander effect in neighbouring uninfected cells. This interferon also activated the accumulation of LDs, which we hypothesis could contribute to this phenomenon. Figure created on BioRender.

IFN-γ, a Type-II IFN has been demonstrated to promote the accumulation of LDs during bacterial infection, therefore, in our model we were interested to understand the relationship of the increased production of IFN and what this might mean for LD accumulation. Interestingly, we saw that a bi-phasic induction of LDs occured following dsRNA viral mimic infection, firstly mediated by EGFR in an IFN independent mechanism, with the second wave, being induced via activation of the JAK/STAT pathway by IFN. Blocking of the Type-I IFN receptor (IFNAR1) significantly inhibited the second wave of LD upregulation, but did not completely abolish it, perhaps indicating that antiviral type-III IFNs may also play a role in this second wave of induction. Interestingly, this phenomenon was not observed following stimulation of cells with dsDNA viral mimics, potentially indicating slightly different biogenesis pathways of LDs, or alternately the co-induction of a negative regulator of LD biogenesis. As we have shown in our recent findings, an upregulation of LDs contributes to a heightened antiviral response, and it is known that virally infected cells generally produce interferon early following infection, prior to potential antagonism of these pathways by viral proteins. This production of interferon is known to upregulate interferon stimulated genes (ISGs) in neighbouring as yet uninfected cells in what is called a ‘bystander effect' to initiate an antiviral environment. Our study now shows that this bystander effect is also likely to involve the upregulation of LDs in concert with the expected upregulation of ISGs, to ensure an effective antiviral state is activated in the local cellular environment. (**Fig. 1**).

The early induction of LDs following a viral infection acts to aid the antiviral host response by enhancing the production of interferon. Overall, our study has positioned LDs as a pivotal organelle in the antiviral innate immune response and represents a paradigm shift in our understanding of the molecular mechanisms which coordinate this response.

